# Problematic smartphone and social media use among undergraduate students during the COVID-19 pandemic: In the case of southern Ethiopia universities

**DOI:** 10.1371/journal.pone.0280724

**Published:** 2023-01-25

**Authors:** Nebiyu Mengistu, Endashaw Habtamu, Chalachaw Kassaw, Derebe Madoro, Wondwosen Molla, Aregahegn Wudneh, Lulu Abebe, Bereket Duko

**Affiliations:** 1 Department of Psychiatry, Dilla University, Dilla, Ethiopia; 2 Department of Midwifery, Dilla University, Dilla, Ethiopia; 3 Curtin School of Population Health, Curtin University, Perth, WA, Australia; The Education University of Hong Kong, HONG KONG

## Abstract

**Background:**

Smartphone and social media use are supposed to be integral parts of university students’ daily lives. More specifically, smartphones and social media are frequently used for communication in daily life during the COVID-19 pandemic. Nonetheless, uninterrupted and persistent use of these technologies may lead to several psychological problems. Even though smartphones and social media were used more frequently during the pandemic, there is no evidence suggesting that the studies were not undertaken in low-income countries, including Ethiopia. Therefore, the current study aimed to assess problematic smartphone use and social media use among undergraduate university students in southern Ethiopia.

**Methods:**

A cross-sectional study was carried out among 1,232 university students using a simple random sampling technique. The Bergen Social Media Addiction Scale and Smartphone Application-Based Addiction Scale were used to collect data on social media and smartphone use, respectively. The Beck Depression Inventory, Generalized Anxiety Assessment Tool, Rosenberg Self-Esteem Scale, and Pittsburg Sleep Quality Index were standardized tools used to measure other independent variables. To identify factors, simple and multiple linear regression analyses were performed. A p-value of 0.05 was used to determine statistical significance.

**Results:**

The overall response rate was 95%. The mean scores for problematic smartphone and problematic social media use were 17 ± 3.3/36 and 12.7 ± 2.2/30, respectively. A linear regression model revealed that being female, first-year students and poor sleep quality were significantly associated with problematic smartphone use. Factors associated with problematic social media use (PSMU) were depression, substance use, and urban residence.

**Conclusions:**

This study identified significant problems with smartphone and social media use among university students. Therefore, it is preferable to provide psychological counselling, educate students about safe, beneficial, and healthy internet use, and focus on recognized high-risk groups in order to give them special attention. It is also preferable to seek counselling about substance use. It is preferable to regularly screen and treat individuals with psychological problems in collaboration with stakeholders.

## Introduction

Coronavirus disease (COVID-19) is a human-to-human communicable respiratory disease caused by a new strain of coronavirus associated with acute respiratory syndrome (SARS-CoV) [1]. After it was originally identified in December 2019 in Wuhan, China, as an emerging respiratory disease, it was abbreviated as COVID-19 [2].

Since the start of the COVID-19 pandemic (on August 20, 2020), 219.3 million people have been infected [3]. Since the outbreak started, Ethiopia’s government has taken a number of measures to stop the spread of COVID-19, including halting schools, enforcing spatial distance, enforcing the use of medical face masks, and banning travel to densely populated areas [4]. As a result of the nationwide actions to stop the spread of Covid-19, schools, institutions, and businesses have shifted to online platforms for virtual learning and employment [1, 4]. This new lifestyle, enforced by staying at home and under quarantine, has brought new challenges socially, economically, physiologically, and psychologically. The COVID-19 pandemic, as well as the accompanying home confinement and social isolation, have heightened fear and an unpleasant mood across society [5–9]. As a result of the pandemic’s various challenging social and psychological changes, daily use of a smart phone and social media becomes a repetitive activity on which every aspect of daily life in every part of the world depends [10, 11].

Problematic smartphone use is a type of behavioral or psychological dependence on mobile devices and is strongly related to other types of excessive use of digital media, like internet addiction disorder. Additionally, a theorized form of psychological or behavioral dependence on social media platforms, sometimes known as internet addiction disorder, is problematic social media use, also referred to as social media addiction or social media overuse [12]. It is a new and attractive subject considered as a behavior-based addiction in recent years [13].

Problematic uses can include preoccupation with mobile communication, excessive money or time spent on mobile phones, and use of mobile phones in socially or physically inappropriate situations such as driving an automobile. Increased use can also lead to adverse effects on relationships or mental or physical health and ensue anxiety if separated from a mobile phone or a sufficient signal. Preschool children and young adults are at the highest risk for problematic smartphone use [14, 15].

Currently, 60% of world population used internet via smart mobile phone and 6.7% of Ethiopian population has used social media [16–18].

Excessive use of smartphones and social media websites, particularly among young adults, is likely to be exacerbated by the essential social-distancing measures of the pandemic [19].

Although smartphones with internet access could be useful for gathering information about the COVID-19 outbreak and communicating with others while under quarantine [16, 20], excessive use of smartphones can lead to maladaptive behaviors such as procrastination and skipping daily tasks, as well as undesirable health repercussions such as sleeplessness and neck/back pain [21].

The majority of studies have found that problematic use of smartphone and social media has a negative impact on one’s physical health and has associations with depression, [22] poor sleep quality, mood changes, and poor health outcomes such as obesity and low self-esteem [23]. Furthermore, the COVID-19 epidemic, as well as the accompanying home quarantine and social-distance measures, have boosted anxieties and negative emotions and felt across society [16]. Several people use smartphones and the internet as coping methods to cope with their emotions. However, the employment of such coping methods may have a number of negative implications, including functional deficits as a result of excessive use [24, 25].

According to a survey conducted in Asian countries like the Middle East, China, Japan, and Bangladesh, the mean scores for problematic smartphone and problematic social media use were 20.8 ± 6.8 and 14.7 ± 4.8 respectively. Younger age, poor sleep, watching television, anxiety, and depression were all associated with problematic smartphone and social media use. Moreover, problematic social media use was associated with being female, urban residence, and alcohol consumption [26–29].

A cross-sectional survey with 425 participants and a median age of 19 years was conducted in South Africa, 59.5% of the participants were young women. Overall, 43.3% had likely depression and 22.4% of the students in a Zambian study reported having a social media addiction. The most problematic smartphone use risk profile is that of a female, under the age of 21, with low self-esteem who lives away from home, making her more vulnerable to problems and also to depression and anxiety [30, 31].

Another cross-sectional survey was conducted on the prevalence and relationship between depression, anxiety, and smart phone addiction among young people in Nigeria. It showed that the prevalence of probable smart phone addiction was 10.2% and 23.4% at the risk of smart phone addiction. Depression, anxiety, financial income level, and being married, using the smart phone for browsing social media and e-mail were the most important predictors of problematic smartphone use [32].

Several studies have been conducted to determine the prevalence of smartphone addiction risks in various countries among college students [33, 34]. However, there were limited studies done in Sub-Saharan African countries that focused on the various aspects of smartphone and social media usage, specifically during the COVID-19 pandemic.

To the best of the investigators’ knowledge, there has been no previous study focusing on problematic smart phone use (PSPU) and problematic social media use (PSMU) among undergraduate university students in Ethiopia during the COVID-19 pandemic. The current study also addressed important contributing factors that stakeholders could control to provide information for students, the need for education about the safe, beneficial, and healthy practices of using social media and smartphones, and the management of psychological issues among students.

Therefore, the aim of this study was to assess problematic smart phone use and problematic social media use and associated factors among undergraduate university students in Ethiopia during the COVID-19 pandemic.

## Methods and materials

### Study design and period

An institution based cross-sectional study was conducted from January 2021 to February 2021 at Dilla and Hawassa Universities.

### Study setting

The study was carried out at the two selected universities in southern Ethiopia, Dilla and Hawassa. The distance between the capital city of Ethiopia and Dilla is 360 kilometers. Additionally, Hawassa University is located in Ethiopia, 278 kilometres south of Addis Ababa. At Dilla University and Hawassa University, respectively, the university had a total of 25,104 and 30,108 undergraduate students throughout the study period.

### Sample size determination, sampling techniques and procedures

The minimum number of sample size required for this study was determined by using the formula to estimate the single population mean, n = (Z alpha/2)2(δ2)/d2, by using the following assumptions: standard deviation (SD) of the mean problematic smart phone score 12.08 [16], a 95% confidence interval (CI) of 1.96 (Z alpha/2 = 1.96), a 1% margin of error (d, 0.01), and a nonresponse rate of 10%. We applied the single population mean formula to give n = (1.96)^2^* (12.08)^2^/ (1)^2^ = 560. By considering a 10% non-response rate and design effects of 2, the final sample size becomes 1,232.

We used a multistage cluster sampling procedure to select a sample of undergraduate students. Initially, three colleges, and two schools were selected by using simple random sampling technique (lottery method) from both universities. In the second stage, the selected colleges and schools were stratified based on the departments.

Dilla University (8 departments) and Hawassa University (11 departments) each have nineteen (19) departments in the selected colleges and schools. All this departments with their level of academic years (batches) were included in this study and the design effect was used. The final sample size was allocated proportionally for each department based on the number of their students with their level academic years (batches). Finally, a simple random sampling technique was used to select participants by using their ID number as a sampling frame.

### Study variables

The dependent variables in this study were Problematic smart phone use (PSPU) and problematic social media use (PSMU) and independent variables were socio-demographic factors (Age, Sex, Religion, Residence, marital status, Academic year, Financial support), Individual level factors (common mode of internet for smartphone and social media access and experience) and Psycho-social and Substance use factors (Depression, Anxiety, sleep quality, Social support, Self-esteem, Peer pressure and current substance use: chat, alcohol, cigarette and others).

### Data collection instruments

The data were collected using self-administered, structured questionnaires. The questionnaire was divided into five(5) sections; It included socio-demographic factors, psycho-social and substance use factors, characteristics of common mode of internet for smartphone and social media access and experience, problematic smart phone use and social media use were used to collect the data. The questionnaire was written in English, translated into Amharic, and then retranslated back into English to ensure consistency.

The dependent variable was measured using the Bergen Social Media Addiction Scale (BSMAS), which was used to assess social media addiction. An advanced psychometric testing (e.g. IRT and network analysis) highlighted that the BSMAS is an easy-to-use, reliable, and valid instrument to assess the social media addiction. This tool was cross-culturally validated instrument with good sensitivity and specificity. It has a Cronbach’s alpha of 0.81. The tool has a five-likert scale ranging from 1 (very rarely) to 5 (very often). It was scored out of 30, and the highest score was considered a problematic social media use [35].

Another outcome variable was measured using the Smartphone Application Based Addiction Scale (SABAS), which was used to assess smart phone addiction. The internal reliability of the scale was good (Cronbach’s alpha 0.88). The SABAS appears to be a valid and reliable ultra-brief tool for a quick and easy assessment of smartphone application-based addiction symptoms. It contains six items and is scored out of 30. All items were rated from 1 (strongly disagree) to 6 (strongly agree). and the highest score was considered a problematic smartphone use [36].

Depression was measured using the Beck Depression Inventory (BDI). It is a standardized instrument that consists of a list of 21 sets of statements. Respondents are asked to choose the statement from each set that most closely describes them or their feelings. Total scores on the BDI were computed by summing the responses to each question. Higher scores indicate depressed mood. Scores were used as a continuous measure or a categorical variable; those scoring > 13 were considered depressed [37].

Anxiety was assessed using a GAD-7 assessment tool and contains seven items that can be responded to on a four-point Likert scale ranging from 0 (*Not at all*) to 3 (*Nearly every day*). The cut-off score ≥10 and had excellent reliability (Cronbach’s alpha = 0.85) [38].

The Rosenberg Self-Esteem Scale was used to assess the level self-esteem. It was a 10 item likert scale scored ranging 1 to 4. The highest score was considered as highest self-esteem [39].

Sleep quality was assed using a 19 item sleep Quality Index (PSQI), a self-report containing seven components of sleep. Each item has 0 to 3 scores. A total score was out 21 and those who scored > 5/21 was considered as poor sleep quality [40].

### Data quality assurance

First, the questionnaire was prepared in English and translated into the local language (Amharic) and then back to English by senior English language expertise to check the accuracy. The questionnaire was pretested at Bulle Horra University among 5% of the calculated sample. During the pretest, the questionnaire was assessed for its clarity, readability, comprehensiveness, accuracy, and optimal time for completing the questioners. The optimal time to complete the questioners and the readability of the items were updated and revised based on the results of the pretest. Two days training were given for the data collectors and supervisors.

### Data analysis and interpretation

The collected data were coded, entered in to EPiDATA version 3.1 and exported to SPSS version 24 for analysis. Simple and multiple linear regression analysis were used to assess the correlates of independent factors with problematic smartphone and social media use with a P-value of <0.25 were considered as candidates of multiple linear regressions. Variables with P- value less than 0.05 were considered as significantly correlated with smart phone and social media use and B coefficient was used to predict the strength of the correlations of variables with smart phone and social media use.

### Ethics approval and consent to participation

The Institutional Review Board (IRB) of Dilla and Hawassa University’s College of Medicine and Health Sciences granted ethical approval. After the purpose and objectives of the study had been informed, oral and written consent was obtained from each study participant before the start of the data collection. To maintain the anonymity and confidentiality of information, similar data collection procedure was in place. And all necessary methods were carried out in accordance with the guidelines of institutional and Declaration of Helsinki.

## Result

### Socio demographic characteristics of respondents

A total of 1,232 study participants, Most of them 800(64.9%) were age 20-24 year old and 750(60.8%) males. Nearly two-thirds 860(69.7%) of them are originated from rural residence and 786(63.8%) of them were senior student (≥2nd year student) *(Table 1)*.

**Table 1:** Socio demographic characteristics of Dilla and Hawassa university undergraduate students, Ethiopia, 2021 (N = 1,232).

Variables	Category	Frequency	Percentage
Age	15-19	394	32.1%
	20-24	800	64.9%
	≥ 25	38	3.1%
Sex	Male	750	60.8%
	Female	482	39.2%
Religion	Orthodox	732	59.5%
	Muslim	226	18.3%
	Protestant	244	19.9%
	Others*	30	2.4%
Marital status	Single	1,080	87.7%
	In relationship	100	8.1%
	Married	30	2.5%
	Others**	22	0.4%
Residence	Rural	860	69.7%
	Urban	372	30.3%
Academic year	Fresh man(1^st^ year)	446	36.2%
	Senior (≥ 2^nd^ year)	786	63.8%
Financial support	From family	1,134	92%
	From relatives	76	6.2%
	Others	22	1.6%

### Characteristics of common mode of internet for smartphone and social media access and experience

Regarding the mode of internet access and its experience, most of the respondents 690(55.9%) were used internet service for above12 months internet use experience and 672(54.6%) of them were used ≥5 hours per day *(Table 2).*

**Table 2:** Characteristics of common mode of internet for smartphone and social media access and experience of Dilla and Hawassa university undergraduate students, Ethiopia, 2021 (N = 1,232).

Internet-use experience (in months)	Never	30	2.5%
	0 to 6	190	15.4%
	6 to 12	322	26.2%
	≥12	690	55.9%
Internet-use per day (in hours)	≤ 5 hours	560	45.4%
	≥5 hours	672	54.6%
Common mode of internet access	Wi-Fi	426	34.5%
	Broadband	158	12.9%
	Mobile internet	648	52.6%

### Psycho-social and substance use characteristics

According to psycho-social and substance use characteristics, out of all respondents 310(25.2%) were developed probable depression, 344(28%) poor sleep quality, 458(37.1%) anxiety and 534(43.4%) low self-esteem. The Current use of substances among 1,232 study participants, 314(25.5%) of them were used alcohol and 418(33.9%) were used khat *(Table 3)*.

**Table 3:** Psycho-social and substance use characteristics of Dilla and Hawassa university undergraduate students, Ethiopia, 2021 (N = 1,232).

Probable depression	Yes	310	25.2%
	No	922	74.8%
Poor sleep quality	Good	888	72.0%
	Poor	344	28.0%
Probable	Yes	458	37.1%
Anxiety	No	774	62.9%
Self-esteem	Low-self esteem	534	43.4%
	High-self esteem	698	56.6%
Current tobacco use	Yes	158	12.6%
	No	1,076	87.4%
Current khat and caffeinated drinks use	Yes	418	33.9%
	No	814	66.1%
Current alcohol use	Yes	314	25.5%
	No	918	74.5%

### Problematic smartphone and social media use and its associated factors among undergraduate students during the COVID-19 pandemic

The mean scores of problematic smartphone use (PSPU) and problematic social media use (PSMU) among undergraduate students at Dilla and Hawassa University were 17 ± 3.3/36 and 12.7 ± 2.2/30, respectively. Multiple linear regression revealed that being female, fresh man students and poor sleep quality were found to be statistically significant with problematic smartphone use *(Table 4)*. Whereas, depression, current substance use, and urban residence were found to be statistically significant with problematic social media use *(Table 5)*.

**Table 4:** Factors associated with problematic smartphone use among Dilla and Hawassa university students, Ethiopia, 2021, (N = 1,232).

Variable	Category	Multiple linear regression			
		B	P-value	95% CI	
Sex	Female	3.474	.000***	2.126	6.73
	Male	1			
Educational status	Fresh man	2.78	.004**	2.47	5.19
	Senior student	1			
Residence	Urban	1.25	0.34	-0.54	1.45
	Rural	1			
Age	15-19	.251	.457	-.317	.949
	20-24	0.32	.256	-.234	.490
	≥ 25	1			
Probable anxiety	No	-3.23	.329	-1.701	-5.205
	Yes				
Poor sleep quality	Yes	5.83	0.00***	2.43	6.89
	No	1			
Self-esteem	Low-self esteem	-1.719	.342	-1.332	-2.106
	High-self esteem				
Current use of khat or caffeinated drinks	No	-1.20	.674	-1.143	0.516
	Yes				
Probable depression	No	-1.71	-.232	-4.01	-1.23
	Yes	1			

**Table 5:** Factors associated with problematic social media use among Dilla and Hawassa university students, Ethiopia, 2021, (N = 1,232).

Variable	Category	Multiple linear regression			
		B	P-value	95% CI	
Sex	Male	-2.46	.334	-5.34	-1.26
	Female	1			
Educational status	Fresh man (1^st^ year)	1			
	Senior student (≥ 2 year)	-5.78	.789	-7.45	-3.94
Residence	Urban	4.54	0.02*	2.45	5.26
	Rural	1			
Age	15-19	.256	.234	-.378	.345
	20-24	0.932	.123	-.290	.490
	≥ 25	1			
Probable anxiety	No	-2.23	.110	-1.690	-3.345
	Yes				
Poor sleep quality	Yes	0.34	0.16	-1.89	0.89
	No	1			
Self-esteem	Low-self esteem	-1.719	.932	-1.10	-3.25
	High-self esteem	1			
Current use of khat or caffeinated drinks	Yes	3.67	.001	1.24	6.41
	No				
Probable depression	Yes	2.45	0.00	1.80	5.23
	No	1			

## Discussion

The use of smart phones and social media has increased substantially all around the world since the era of the pandemic [16]. According to the current study findings, more than half of the respondents, scored above the mean for problematic smart phone use, while one third of them scored above the mean for problematic social media use. Females, freshmen students, and poor sleep quality were shown to be characteristics linked with problematic smartphone use, whereas depression, substance use, and urban living were found to be factors associated with problematic social media use (PSMU). This study finding was lower than the studies done in Lebanon [41], Zambia [42] and Bangladesh [43]. This variation may be due to the accessibility, knowledge and attitude difference towards smart phone use and social media use.

This study found that being females increase the problematic smart phone use score by 3.474 unit as compared to their counterpart which was supported by the studies conducted in Jordan [27], china [28], and japan [29]. The possible justification for this strong association could be due to the fact most female respondents accessed the internet on their smartphones to search for relevant information [28], and the majority of them said they used their smartphones for accessing academic information, reading news, entertainment, and listening to music.

Freshman students were showed an increment on problematic smart phone use score by 2.78 units as compared to senior students (≥ 2^nd^ year students). The finding was similar with the studies conducted in Ghana [44], China [45]. This might be due to the fact that smartphones provide the ability to get answers really fast. In some situations, a student may not ask for clarification to a question he or she has in an open classroom because they can use their smartphone to get the answer they’re looking for. Audio and video can bring learning to life [46]. And another possible explanation is that new campus students encounter problems such as being separated from their families, adjusting to a new setting, making new acquaintances, and learning a new culture, all of which encourages them to stay glued to their smartphones [47].

Those respondents with poor sleep quality showed that a 5.83 unit increases in problematic smart phone use score as compared to their challengers which was supplemented by Saudi Arabia [48], Belgium [49] and United states [50]. This may be explained due to the fact that smart phone causes abnormal sleep inducing physiological process such as melatonin production associated with difficulty of sleep initiation and maintenance [51].

According to the current study finding result, respondents who had depression increase 2.45 units on problematic social media use which was similar with the study finding done in United states [52], Nigeria [30], South Africa [31]. The possible reason for this may be that people who are depressed may be more likely to utilize problematic social media platforms like Facebook, Twitter, and YouTube. A depressive state can make it difficult to manage stress, and subjects may turn to social media to distract themselves. Because of this, students who experience depressed symptoms frequently turn to social media to connect with distant friends and find temporary comfort, which leads to their addiction to the social media use [53, 54].

Those respondents with current substance use history had increase on problematic social media score by a 3.67 units as those with no current substance use history. This finding was supported by the studies done in Norway [55], Canada [56] and united states [57].

The drug’s mechanism of action causes users to search for entertaining content online while they are intoxicated or going through withdrawal. This could be explained by the biological impact of the drugs on the brain, which makes them stimulants of the central nervous system that can improve focus and alertness, uplift the mood, increase motivation for work, and have addictive or compulsive effects that are also linked to symptoms of problematic internet use. As a result, many individuals may be readily persuaded or driven to use the internet [57].

Those with urban residence had a problematic social media use score increase by a 4.54 unit as compared with those in rural area which was similar with the study finding in Bangladesh [43], Hungary [58]. The possible reasons explained due to the fact that living in a city gave you access to a variety of social media and technology, which you can utilize in your day-to-day activities.

### Implications

Since the pandemic era, social media and smartphone usage have grown significantly all across the world. Prior to the discovery of the covid-19 virus, it was not widely used in Sub-Saharan African countries. Users of smartphones have more options because they can improve their capabilities by downloading various mobile applications. Many university students’ life revolve around their smartphones. However, it can be harmful to only have access to a smartphone and social media without specific directed educational activities. That is, using technology in an excessive or problematic way can cause a variety of psychological and mental conditions, such as anxiety, depression, substance abuse, and poor sleep quality. However, there was little information available on the problems with social media and problematic smartphone use during the COVID-19 pandemic. In order to reduce misconceptions, stakeholders like psychiatrists and psychologists should improve psychoeducation by addressing problematic smartphone and social media use. Governments should also provide institutional-based mental health services in light of the significance of psychological education in addressing problematic smartphone and social media use among university students.

### Limitation

The current study was limited to assessing students’ learning behaviors which could be modifiable determinants in problematic smart phone and social media use. Another limitation of this study is that, due to the cross-sectional nature of the study design, it does not show any cause-effect relationship. There may be a social desirability bias, where students may not have provided exact web browsing statistics in order to impress the investigator.

## Conclusion

There was significant, problematic smart phone and social media use among university students. This study revealed the psychosocial and sociodemographic characteristics that require treatment. The results suggest that in order to combat the expected increase in smart phone and social media use, it is better to counsel on substance use and its effects, educate on safe, valuable, and healthy smartphone or internet use, and give special emphasis to identified high-risk groups. Additionally, students need to be educated about safe, valuable, and healthy internet use. Furthermore, it is better to have routine screening and treatment of individuals having such psychological problems through collaboration with stakeholders.

## List of abbreviations

BDI: Beck Depression Inventory
BSMAS: Bergen Social Media Addiction Scale
CI: Confidence Interval
EpiData: Epidemiological Data
LAMIC: Low- and Middle-Income Countries
PSMU: Problematic Social Media Use
PSPU: Problematic Smartphone Use
SABAS: Smartphone Application Based Addiction Scale
SPSS: Statistical Package for Social Science
SSA: Sub-Saharan Africa
UNICEF: United Nations Children’s Fund
WHO: World Health Organization
